# The impact of non-invasive brain stimulation and digital intervention on social cognitive therapy in autism spectrum disorder: a systematic review and network meta-analysis

**DOI:** 10.3389/fpsyt.2026.1877176

**Published:** 2026-08-31

**Authors:** Juhao Hou, Yi Fang, Yue Wang, Shilin Wen

**Affiliations:** 1 Capital University of Physical Education And Sports, Bei Jing, China; 2 North China Institute of Aerospace Engineering, Lang Fang, He Bei, China

**Keywords:** autism spectrum disorder, network meta-analysis, social cognition, transcranial direct current stimulation, transcranial magnetic stimulation, virtual reality

## Abstract

**Introduction:**

To evaluate the relative efficacy of non-invasive brain stimulation (NIBS) and digital interventions for improving social cognition in autism spectrum disorder (ASD).

**Methods:**

A systematic search of PubMed, Embase, Web of Science, Cochrane, CNKI to February 2026 yielded 50 randomized controlled trials (RCTs). A Bayesian network meta-analysis was performed using R, ranking interventions via Surface Under the Cumulative Ranking curve (SUCRA) and assessing evidence certainty with Confidence in Network Meta-Analysis (CINEMA).

**Results:**

Across the included trials (mean age range: 2.54 to 20.80 years), among 9 interventions, transcranial direct current stimulation (tDCS) emerged as optimal (SUCRA = 88.24%), significantly outperforming treatment as usual (TAU) (SMD=-1.69, 95% CrI: -2.67, -0.70). This was followed by high-frequency repetitive transcranial magnetic stimulation (HF-rTMS) (84.38%, SMD=-1.60, 95% CrI: -2.32, -0.89), low-frequency repetitive transcranial magnetic stimulation (LF-rTMS) (74.22%, SMD=-1.36, 95% CrI: -1.77, -0.96), and theta-burst stimulation (TBS) (64.21%, SMD=-1.19, 95% CrI: -2.02, -0.35); all significantly outperformed TAU. Standalone digital interventions ranked lower. Meta-regression showed no significant age moderating effect.

**Conclusions:**

tDCS is the most efficacious standalone intervention for ASD social cognitive deficits. We recommend tDCS or TBS clinically and advocate a synergistic paradigm integrating NIBS neural priming with digital training. However, future large-scale, rigorous RCTs are required to further validate both these standalone modalities and the proposed combined protocols.

**Systematic review registration:**

https://www.crd.york.ac.uk/PROSPERO/view/CRD420261337491, identifier CRD420261337491.

## 1 Introduction

Navigating the profound heterogeneity of Autism Spectrum Disorder (ASD) remains a formidable challenge in clinical research. While the condition is defined by a complex interplay of restricted behaviors and pervasive communication deficits, it is the profound impairment in social cognition that frequently emerges as the most disruptive clinical manifestation (1). We know that higher-order processing capacities—such as facial recognition, emotion comprehension, and joint attention—are not merely cognitive milestones; they form the very bedrock of interpersonal connection and meaningful social integration (2). The functional consequences of these impairments are undeniable. They not only isolate individuals but also place a huge and long-term strain on families and the broader healthcare infrastructure. To manage these deficits, current clinical practice—our Treatment as Usual (TAU)—leans almost entirely on conventional behavioral and educational interventions. However, the reality of everyday rehabilitation paints a far more complicated picture. The most immediate friction is often logistical. The traditional treatment plans are lengthy and rely on a limited number of professionals, resulting in systemic bottlenecks that prevent vulnerable groups from receiving timely treatment (3). However, the challenges we have observed in practice are far more than just the issue of resource allocation. The core of autism spectrum disorder (ASD) lies in its significant neurobiological diversity - and the standard, single-type behavioral patterns simply cannot address this reality. When general methods conflict with highly individualized neurological characteristics, clinical efficacy is bound to stagnate. It is precisely this underlying mismatch, rather than a mere lack of patient effort, that transforms long-term compliance from a therapeutic goal into a pervasive practical hurdle (4). Therefore, breaking through the limitations of the traditional rehabilitation model and seeking new auxiliary or alternative therapies, especially those that combine clear neurophysiological goals with high ecological validity, has become an urgent and crucial task in contemporary clinical research on ASD.

This pressing need has naturally driven contemporary research toward the intersection of advanced engineering and a more nuanced understanding of the “social brain” network, crystallizing into two major categories of emerging interventions with substantial clinical potential. The first of these confronts physiological bottlenecks directly through non-invasive brain stimulation (NIBS). By utilizing techniques like repetitive transcranial magnetic stimulation (rTMS) and transcranial direct current stimulation (tDCS), researchers are attempting to bypass the protracted learning curves of traditional therapy to actively modulate cortical excitability, thereby inducing long-term neuroplasticity. What makes this neuromodulatory approach particularly compelling from a clinical perspective is its target specificity. Emerging data suggests that precisely stimulating distinct nodes of the social cognition network does more than just alter local activity; it actively facilitates the remodeling of aberrant neural circuits (5). We see this vividly in practice: applying high-frequency rTMS over the dorsolateral prefrontal cortex (DLPFC) acts as a functional catalyst for the frontal system, translating behaviorally into a sharper, more reliable recognition of complex facial micro-expressions (6). Shift the stimulation target to the right temporoparietal junction, however, and the therapeutic effect uniquely pivots to enhance affective resonance, directly bridging the gap between localized neural activation and real-world empathetic capacity (7). On the other hand, digital interventions—encompassing immersive virtual reality (VR), embodied social robots, and two-dimensional interactive software—provide a behavioral intervention paradigm characterized by high ecological validity. Taking VR as an example, this technology can construct highly structured, safe, and reproducible immersive social environments for patients. This interactive modality not only significantly mitigates the trial-and-error costs and anxiety thresholds associated with real-world social interactions but also effectively enhances joint attention and social motivation through multisensory feedback mechanisms (8).

Yet, for all the physiological elegance and clinical promise of these emerging modalities, translating this potential into frontline practice reveals a significant evidence gap. When we critically examine the current literature, it becomes evident that the field remains heavily siloed in traditional pairwise designs—predominantly evaluating an isolated therapy against a sham baseline or standard TAU. While this establishes basic efficacy, it falls short of addressing the complexities of clinical decision-making. Faced with a rapidly expanding menu of treatments, clinicians and families are often left navigating without a compass due to a glaring absence of direct, head-to-head comparative data—whether attempting to evaluate entirely distinct intervention categories or parse out specific protocols within the same family of treatments. This void is a substantial barrier to clinical translation. It is a tangible barrier that actively stalls the momentum of precision rehabilitation, keeping us from formulating the kind of truly personalized, evidence-based intervention hierarchies that the highly heterogeneous ASD population urgently requires.

As an advanced methodological framework in evidence-based medicine, network meta-analysis (NMA) employs rigorous statistical modeling to quantitatively synthesize both direct and indirect comparative evidence. Crucially, in the absence of direct head-to-head trials, NMA facilitates the comprehensive evaluation and probabilistic ranking of the relative efficacy of multiple interventions within a unified evidence network. Therefore, the present study aims to comprehensively evaluate and compare the relative efficacy of NIBS—specifically low-frequency and high-frequency rTMS, tDCS, and theta-burst stimulation (TBS)—and digital interventions, including immersive virtual reality (VR), embodied social robots, and two-dimensional interactive software, in improving social cognition in ASD. By delineating the efficacy hierarchy of these diverse interventional strategies, this study seeks to resolve the existing clinical decision-making impasse and provide high-quality, evidence-based support for the optimization of ASD intervention protocols.

## 2 Methods

This systematic review and network meta-analysis were conducted and reported in strict accordance with the PRISMA-NMA guidelines. The study protocol was prospectively registered in the PROSPERO database (ID: CRD420261337491).

### 2.1 Search strategy

Two investigators independently conducted a comprehensive literature search across the following electronic databases: PubMed, Embase, Web of Science, the Cochrane Library, and the China National Knowledge Infrastructure (CNKI), spanning from their respective inceptions to February 2026. To ensure literature saturation, the reference lists of all included trials and relevant review articles were manually cross-checked to identify any eligible studies potentially missed by the electronic database searches. No language restrictions were imposed during the search process. The search strategy employed a rigorous combination of Medical Subject Headings (MeSH) and free-text keywords. Core search terms encompassed “Autism Spectrum Disorder,” “Social Cognition,” “Non-invasive Brain Stimulation,” “Transcranial Magnetic Stimulation,” “Transcranial Direct Current Stimulation,” “Virtual Reality,” and “Robotics”.

### 2.2 Inclusion and exclusion criteria

Inclusion Criteria: ① Population: individuals with a confirmed diagnosis of ASD across all age groups and genders, verified by standardized clinical criteria like the Autism Diagnostic Observation Schedule (ADOS); ② Intervention: non-invasive brain stimulation (LF-rTMS, HF-rTMS, tDCS, or TBS) or digital therapies (immersive VR, embodied social robots, or 2D interactive software); ③ Comparator: utilization of sham interventions or treatment as usual (TAU) for the control cohort; ④ Outcomes: availability of post-treatment quantitative data on social cognition or core interactive functions, measured by established instruments like Childhood Autism Rating Scale (CARS), Autism Behavior Checklist (ABC), or Social Responsiveness Scale (SRS); ⑤ Study Design: randomized controlled trials (RCTs).

Exclusion Criteria: ① involved non-ASD populations; ② utilized experimental interventions other than the predefined NIBS or digital modalities; ③ were non-original research articles (e.g., systematic reviews, case reports, editorials); ④ provided incomplete or unextractable primary data, and the core data remained inaccessible even after corresponding with the original authors; ⑤ failed to report relevant social cognitive outcome measures; ⑥ employed non-RCT experimental designs.

### 2.3 Data extraction and risk of bias assessment

Data extraction was performed independently by two investigators using a pre-piloted, standardized data collection form. The extracted information encompassed: study characteristics (first author and publication year), participant demographics (sample size and age), specific intervention protocols (modality, frequency, and duration), adverse events, and clinical outcome data (baseline and post-intervention scale scores). Specifically, for the quantitative synthesis, our analyses were based exclusively on post-intervention final values (means and standard deviations). All extracted data represent measurements obtained immediately following the completion of the intervention protocols. For studies reporting continuous outcomes as medians and interquartile ranges (IQRs), the sample mean and standard deviation (SD) were rigorously estimated using the validated algorithm proposed by Wan et al. (9), thereby ensuring the robust synthesis of effect sizes. The risk of bias for each included randomized controlled trial was independently evaluated by the two investigators using the revised Cochrane risk-of-bias tool for randomized trials (RoB 2). Any discrepancies arising during the data extraction or quality assessment processes were resolved through discussion to reach a consensus, or, if necessary, via adjudication by a third independent reviewer.

### 2.4 Statistical analysis

A Bayesian network meta-analysis (NMA) was conducted using R software (version 4.5.2) to quantitatively synthesize the included data. Given the diverse assessment scales employed across studies to measure social cognition in patients with ASD, the standardized mean difference (SMD) along with its corresponding 95% credible interval (CrI) was utilized as the pooled effect size. Network plots were generated to visually map the direct and indirect comparative relationships among the various interventions and control conditions. A random-effects model was employed to pool the evidence. Global between-study heterogeneity across the evidence network was primarily quantified using the estimated between-study variance (*τ*^2^). Specifically, the posterior median of *τ*^2^ and its 95% credible interval (CrI) were extracted from the Bayesian random-effects model utilizing standardized mean differences (SMDs) to account for variations in scale properties.

For the Bayesian model parameterization, Markov Chain Monte Carlo (MCMC) simulations were executed with four parallel chains. Each chain consisted of 20,000 iterations, with the initial 5,000 iterations discarded as the burn-in period to ensure model convergence. A thinning interval of 10 was applied to mitigate sample autocorrelation. Furthermore, the underlying assumptions of the evidence network were rigorously evaluated. To assess global consistency, an unrelated mean effects (UME) model was constructed, and its goodness-of-fit was compared against the standard consistency model utilizing the Deviance Information Criterion (DIC) and residual deviance (Dbar). Comparable values between the two models indicated robust global consistency. Local consistency was evaluated using the node-splitting method to detect discrepancies between direct and indirect evidence within closed loops, with a P > 0.05 denoting no significant inconsistency.

To determine the relative superiority of the various interventions in improving social cognition, the Surface Under the Cumulative Ranking curve (SUCRA) was calculated; a higher SUCRA value indicates a greater probability of a specific intervention being the optimal therapy. Finally, comparison-adjusted funnel plots coupled with Egger’s test were employed to detect potential publication bias and small-study effects. Additionally, a network meta-regression analysis incorporating the mean age of participants was conducted to explore the potential moderating effect of age on treatment efficacy.

### 2.5 Assessment of publication bias and certainty of evidence

To investigate potential publication bias and small-study effects across the included trials, a visual inspection of comparison-adjusted funnel plots was initially conducted to assess contour symmetry. This visual assessment was subsequently corroborated by Egger’s regression test, with a P < 0.05 considered indicative of significant asymmetry. Furthermore, to establish a rigorous level of confidence in the interpreted relative treatment effects, the Confidence in Network Meta-Analysis (CINEMA) framework was systematically applied to evaluate the overall certainty of evidence for all primary pairwise comparisons.

## 3 Results

### 3.1 Literature search results

**Figure 1:**
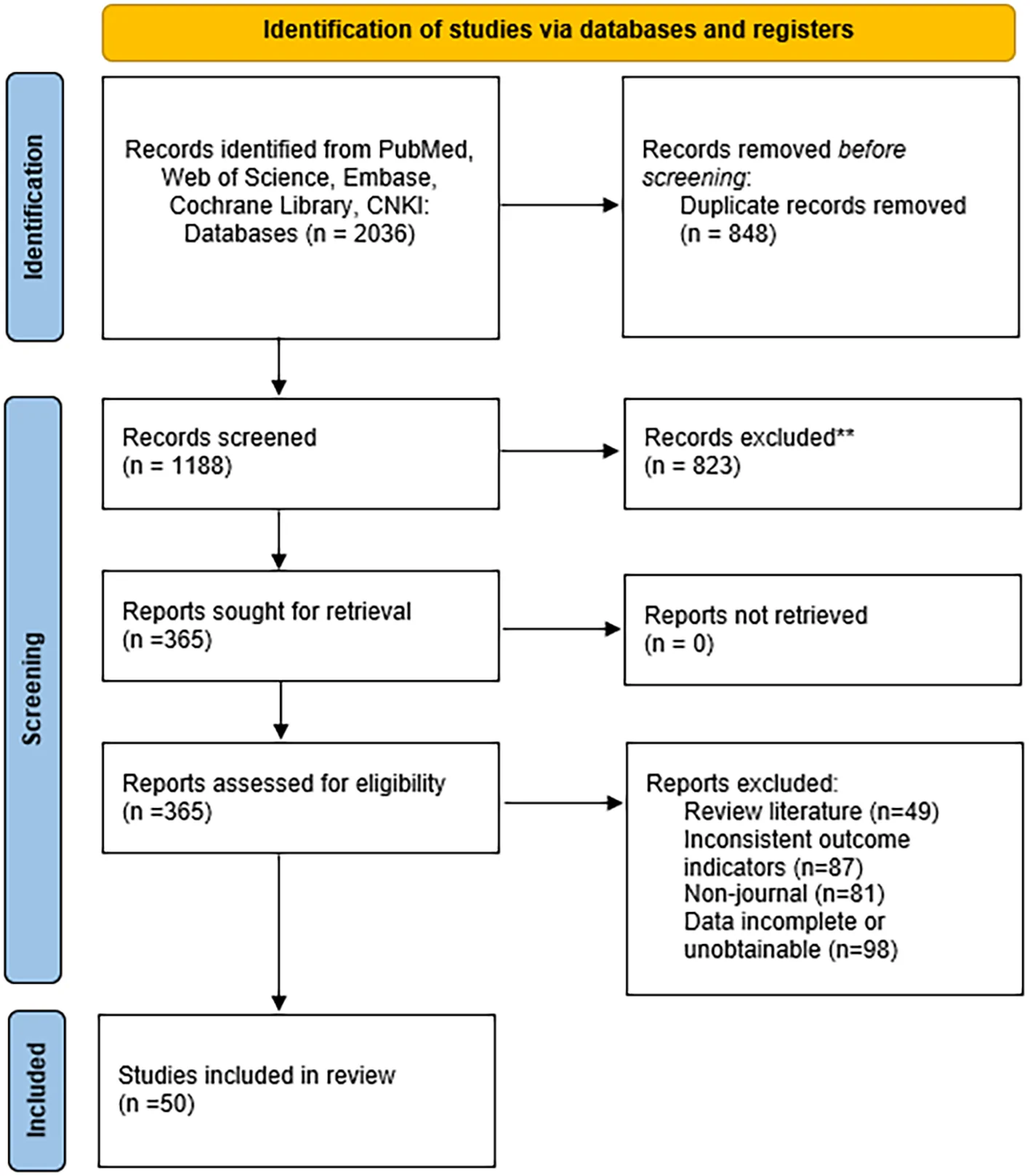
PRISMA flow diagram of the study selection process.

The literature selection process was conducted in strict accordance with the PRISMA flow diagram (**Figure 1**). Systematic searches across both Chinese and English databases initially yielded 2,036 relevant records. Following the removal of 848 duplicates, the titles and abstracts of the remaining 1,188 articles were screened, resulting in the exclusion of 823 studies that failed to meet the predefined inclusion criteria. Subsequently, 365 articles underwent comprehensive full-text evaluation for eligibility. After the exclusion of 315 articles for failing to satisfy the eligibility requirements, a total of 50 randomized controlled trials were identified as eligible and included in the final network meta-analysis.

### 3.2 Characteristics of included studies

The 50 eligible studies comprised a total of 3,191 patients with a definitive diagnosis of autism spectrum disorder (ASD). The participants were predominantly children and adolescents, with an overall mean age of 7.09 years (mean age range across included studies: 2.54 to 20.80 years). This systematic review focused on non-pharmacological interventions for social cognition in ASD, yielding nine discrete nodes for the network meta-analysis. Regarding non-invasive brain stimulation (NIBS), the evidence base included 14 trials of low-frequency repetitive transcranial magnetic stimulation (LF-rTMS) (10–23), four trials of high-frequency rTMS (HF-rTMS) (24–27), nine trials of tDCS (28–37), and six trials of theta-burst stimulation (TBS) (37–42). Additionally, digital interventions encompassed seven trials of immersive virtual reality (VR) (43–49), five trials of embodied social robots (50–54), and five trials of 2D screen-based interactive software (55–59). Control conditions were primarily categorized as treatment as usual (TAU) or sham stimulation plus TAU; for clarity, the latter was designated as “Sham.” Comprehensive details of the included studies—including first authors, publication years, sample sizes, intervention parameters, and specific social cognition assessment scales—are systematically presented in **Supplementary Tables 1**, **2**.

While all participants had a definitive diagnosis of ASD, detailed reporting on co-occurring additional disabilities (such as intellectual disability or attention-deficit/hyperactivity disorder) was generally absent across the primary literature, limiting further subgroup analysis on these comorbidities.

### 3.3 Risk of bias assessment

**Figure 2:**
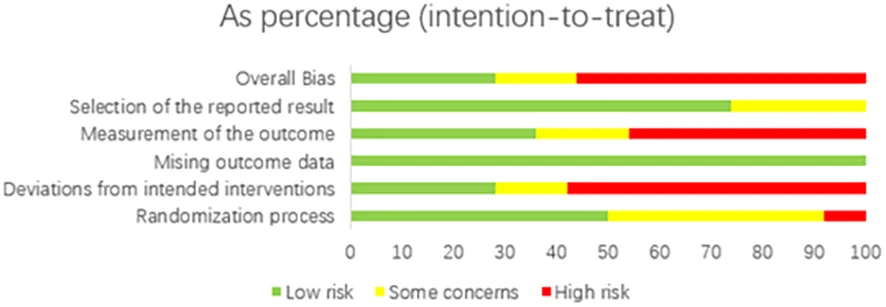
Risk of bias summary plot.

The methodological quality of the 50 included randomized controlled trials was independently evaluated by two investigators using the Cochrane Risk of Bias tool (RoB 2), with results indicating a moderate-to-high overall quality. The majority of the studies exhibited a low risk of bias in the domains of random sequence generation and completeness of outcome data. However, a common challenge was identified in the “blinding of participants and personnel” domain, particularly within digital intervention groups. Due to the highly interactive nature inherent in immersive VR and embodied social robots, implementing strict double-blind protocols presented significant practical difficulties—a recognized inherent limitation in non-pharmacological rehabilitation trials. Nevertheless, most studies maintained robust blinding of outcome assessors, thereby minimizing potential measurement bias to the greatest extent possible. The overall risk of bias summary and granular assessments for individual studies are presented in **Figure 2**, **Supplementary Figure 1** respectively.

### 3.4 Results of the network meta-analysis

#### 3.4.1 Overall network meta-analysis

**Figure 3:**
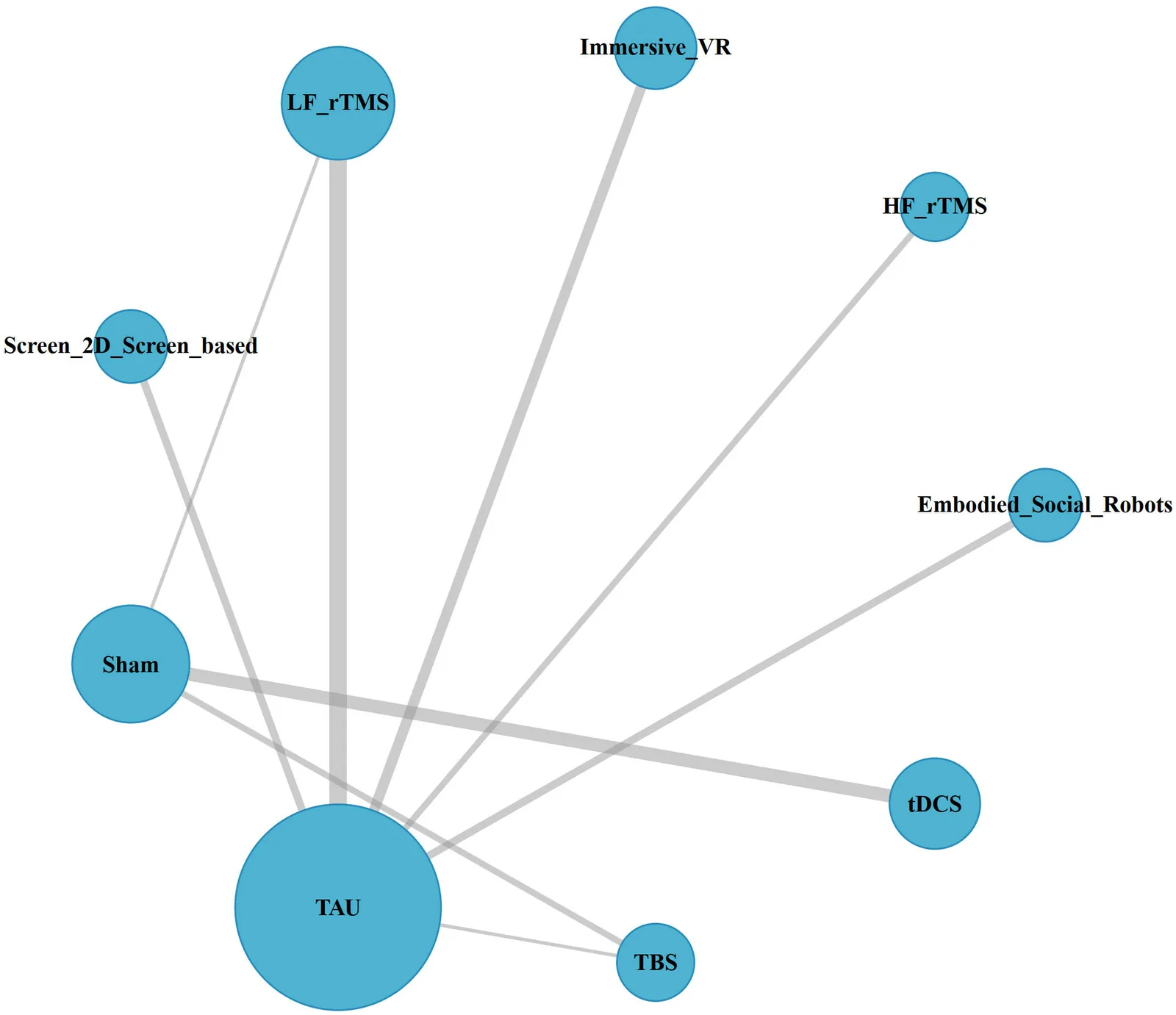
Network of direct and indirect comparisons for NIBS and digital interventions on social cognition in ASD.

The study included a total of 50 trials involving nine distinct interventions. The results of the Bayesian network meta-analysis demonstrated a moderate-to-high level of global heterogeneity, with an estimated between-study variance of *τ*^2^ = 0.55 (95% CrI: 0.3336, 0.9604). This variance aligns with the anticipated clinical heterogeneity inherent in diverse non-pharmacological interventions for ASD, further validating our application of a random-effects model. Furthermore, the Deviance Information Criterion (DIC) was employed to evaluate the goodness-of-fit of the model. For the consistency model, DIC = 95.57, which was similar to the inconsistency model with DIC = 95.3. The difference between the two is less than 5, indicating that the data satisfied the global consistency assumption, and the consistency model adequately fitted the current data (**Figure 3**). Furthermore, the diagnostic trace plots demonstrated that the Markov Chain Monte Carlo (MCMC) chains were well-mixed and stationary with no discernible trend deviations, and the posterior density plots exhibited smooth, unimodal distributions, which further confirmed the stability and reliability of the model parameter estimates (**Supplementary Figures 2**, **3**). Within the network plot, each node represents a specific intervention type, with its size proportional to the total sample size for that intervention. The connecting lines indicate direct comparisons, and their thickness corresponds to the number of studies contributing to each comparison. Among the included trials, 16 studies used the Childhood Autism Rating Scale (CARS); 12 used the Social Responsiveness Scale (SRS); 9 used the Autism Treatment Evaluation Checklist (ATEC); 5 used the Autism Behavior Checklist (ABC); 2 used the Social Interaction subscale of the Psychoeducational Profile-Third Edition (PEP-3); 2 used the Autism Diagnostic Observation Schedule (ADOS); 1 used the Vineland Adaptive Behavior Scales, Second Edition (VABS-II); 1 used the Screening Tool for Autism in Toddlers and Young Children (STAT); 1 used the Communication and Symbolic Behavior Scales (CSBS); and 1 used the Test of Emotion Comprehension (TEC). For positively valenced scales, the study required their means to be multiplied by -1 prior to calculating the standardized mean differences (SMDs), while the standard deviations (SDs) were maintained as absolute values, ensuring uniformity in the directionality of all SMDs.

**Figure 4:**
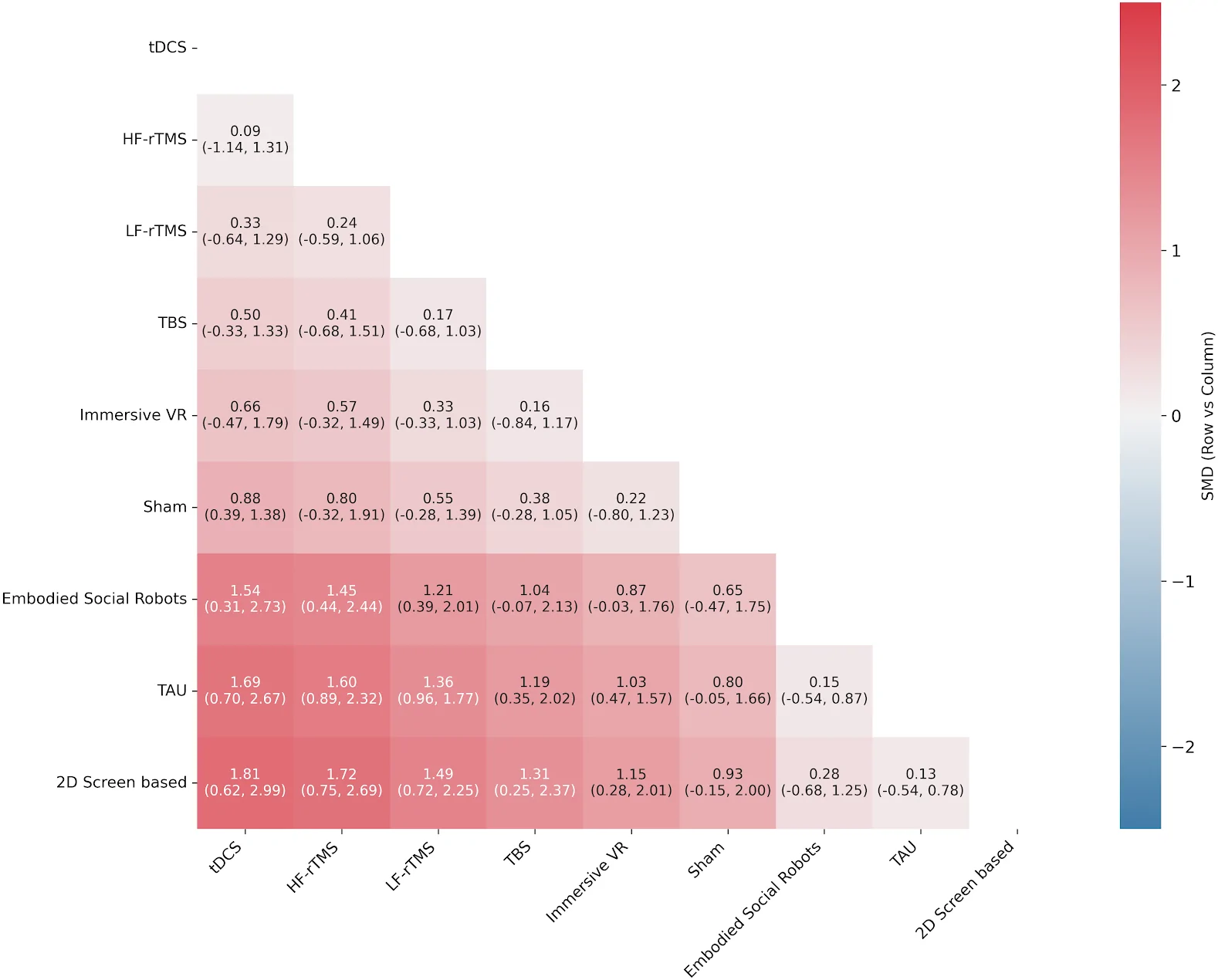
League table of all pairwise comparisons for the included interventions.

**Figure 4** indicates that tDCS demonstrated a statistically significant superiority over treatment as usual (TAU) (SMD = -1.69, 95% CrI: -2.67, -0.70). Similarly, HF-rTMS was significantly superior to TAU in reducing symptom scores (SMD = -1.60, 95% CrI: -2.32, -0.89). Furthermore, LF-rTMS was significantly superior to TAU (SMD = -1.36, 95% CrI: -1.77, -0.96). TBS was significantly superior to TAU (SMD = -1.19, 95% CrI: -2.02, -0.35). Immersive VR was significantly superior to TAU (SMD = -1.03, 95% CrI: -1.57, -0.47). Interventions involving 2D screen-based software and embodied social robots failed to show a statistically significant improvement compared to TAU (**Supplementary Figure 4**). Additionally, following a network meta-regression analysis adjusting for age, the relative efficacy among the various therapies did not change significantly (B = -0.0431, 95% CrI: -1.05, 0.91).

#### 3.4.2 Cumulative ranking probabilities

**Figure 5:**
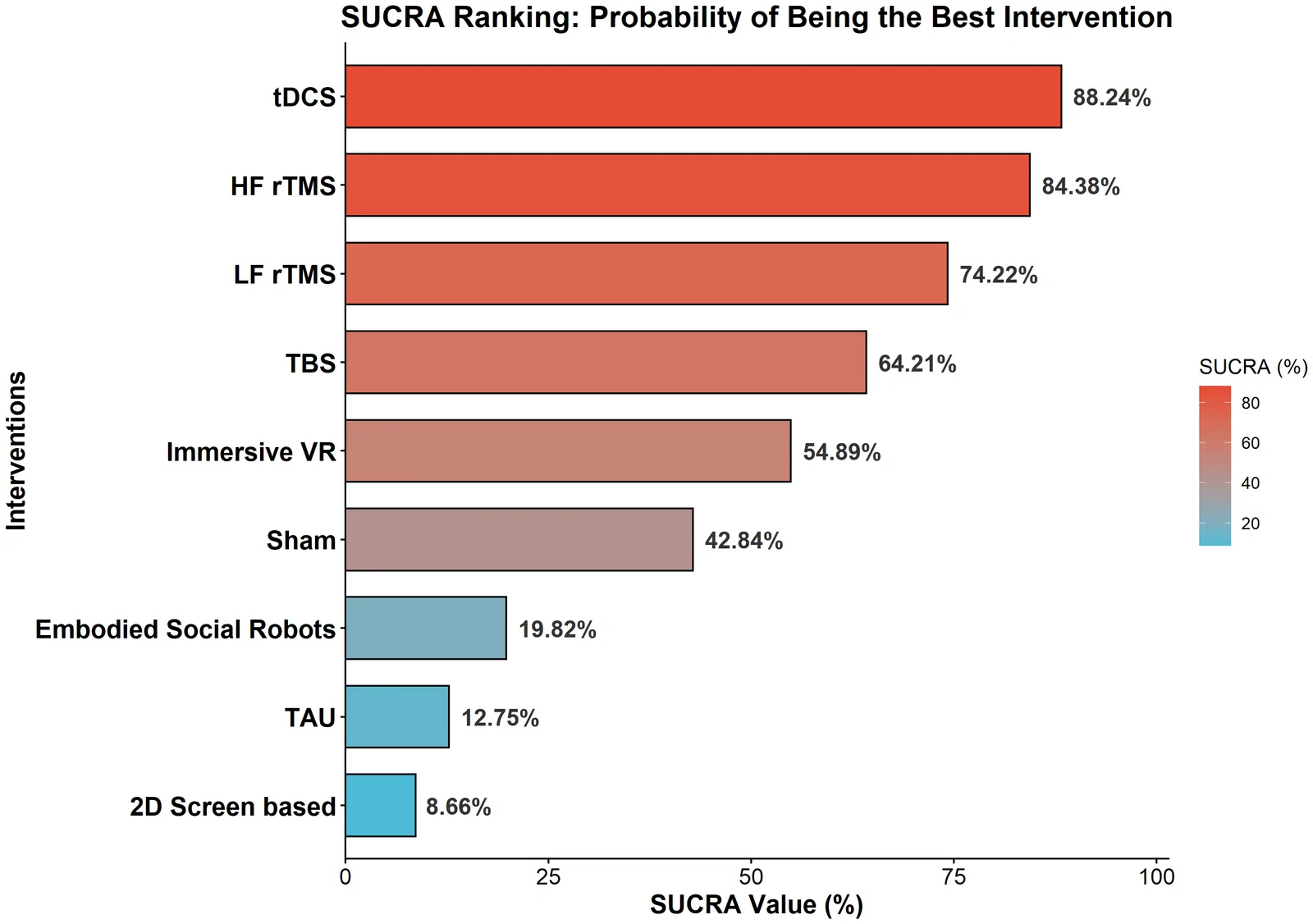
Cumulative ranking probability plots for the evaluated interventions.

The surface under the cumulative ranking curve (SUCRA) was utilized to rank the relative efficacy of the nine interventions (**Figure 5**; **Table 1**). tDCS was ranked as the most effective intervention (SUCRA = 88.24%). This was closely followed by rTMS technologies, including HF-rTMS (SUCRA = 84.38%), LF-rTMS (SUCRA = 74.22%), and TBS (SUCRA = 64.21%), all of which demonstrated favorable clinical efficacy. Immersive VR (SUCRA = 54.89%) exhibited moderate efficacy. In contrast, Sham (SUCRA = 42.84%), embodied social robots (SUCRA = 19.82%), and TAU (SUCRA = 12.75%) yielded inferior therapeutic outcomes. 2D screen-based interventions (SUCRA = 8.66%) ranked at the bottom of the network, representing the least effective protocol for improving this specific clinical outcome.

**Table 1:** SUCRA values and cumulative ranking probabilities of interventions for social cognition.

Intervention	SUCRA percentage
tDCS	88.24%
HF-rTMS	84.38%
LF-rTMS	74.22%
TBS	64.21%
Immersive VR	54.89%
Sham	42.84%
Embodied Social Robots	19.82%
TAU	12.75%
2D Screen based	8.66%

### 3.5 Consistency and heterogeneity analysis

**Figure 6:**
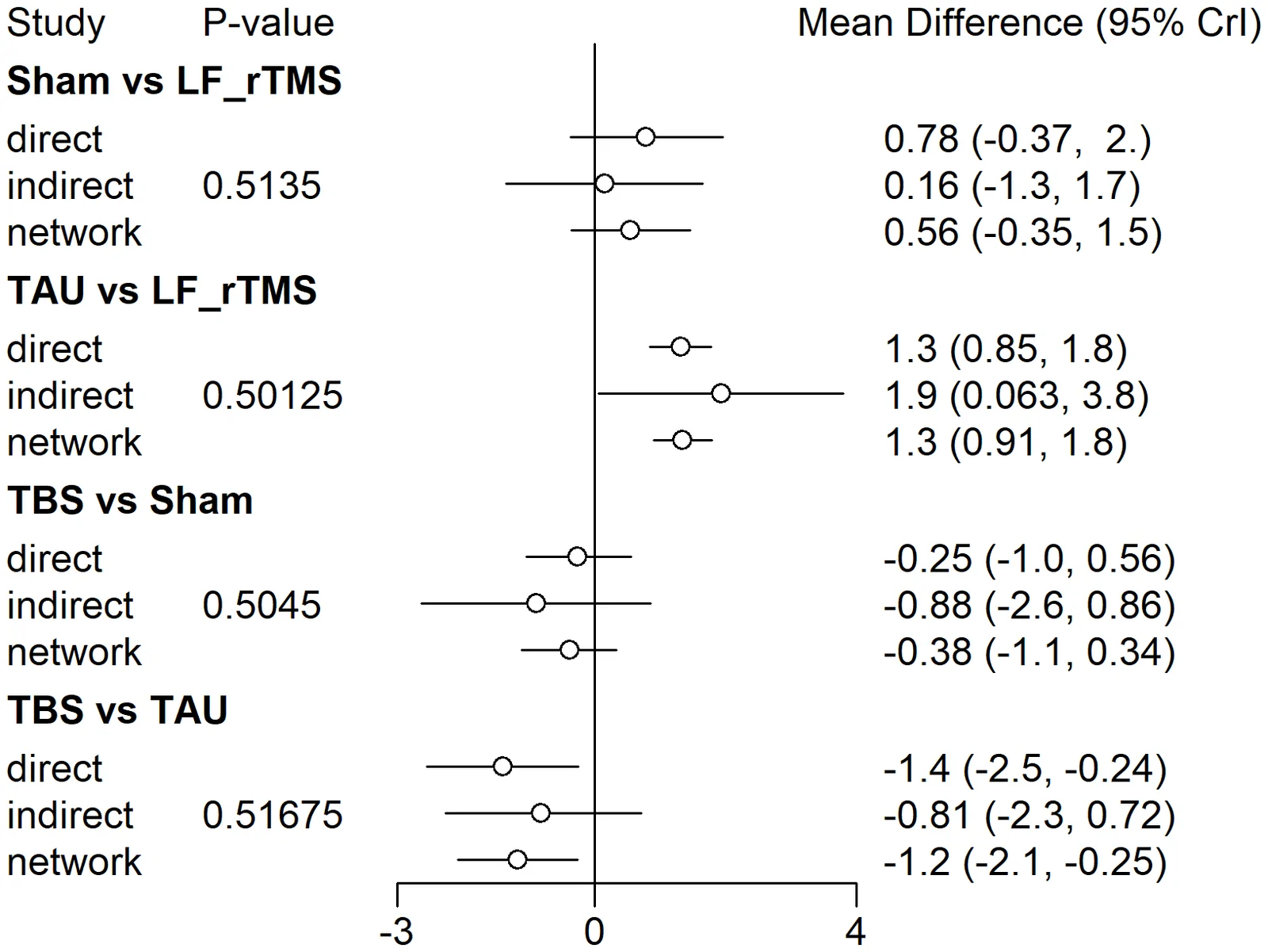
Node-splitting forest plot.

The global consistency of the evidence network was evaluated, revealing no significant inconsistency (P = 0.474 > 0.05). Furthermore, the consistency model exhibited the optimal fit to the data, yielding Dbar = 53.12 and pD = 42.50. To rigorously examine the local consistency between direct and indirect evidence, the node-splitting method was employed. The analysis identified multiple closed loops involving LF-rTMS, TBS, Sham, and TAU: LF-rTMS vs. Sham, LF-rTMS vs. TAU, TBS vs. Sham, and TBS vs. TAU. The results indicated no statistically significant discrepancies between the direct and indirect estimates for any of the specified comparisons (P = 0.514, P = 0.501, P = 0.505, and P = 0.517) (**Figure 6**). Moreover, the point estimates derived from direct and indirect comparisons were highly analogous across all closed loops. These findings robustly confirm that the current data strictly satisfied the consistency assumption, thereby further validating the overall reliability of the network meta-analysis results.

### 3.6 Publication bias and certainty of evidence

**Figure 7:**
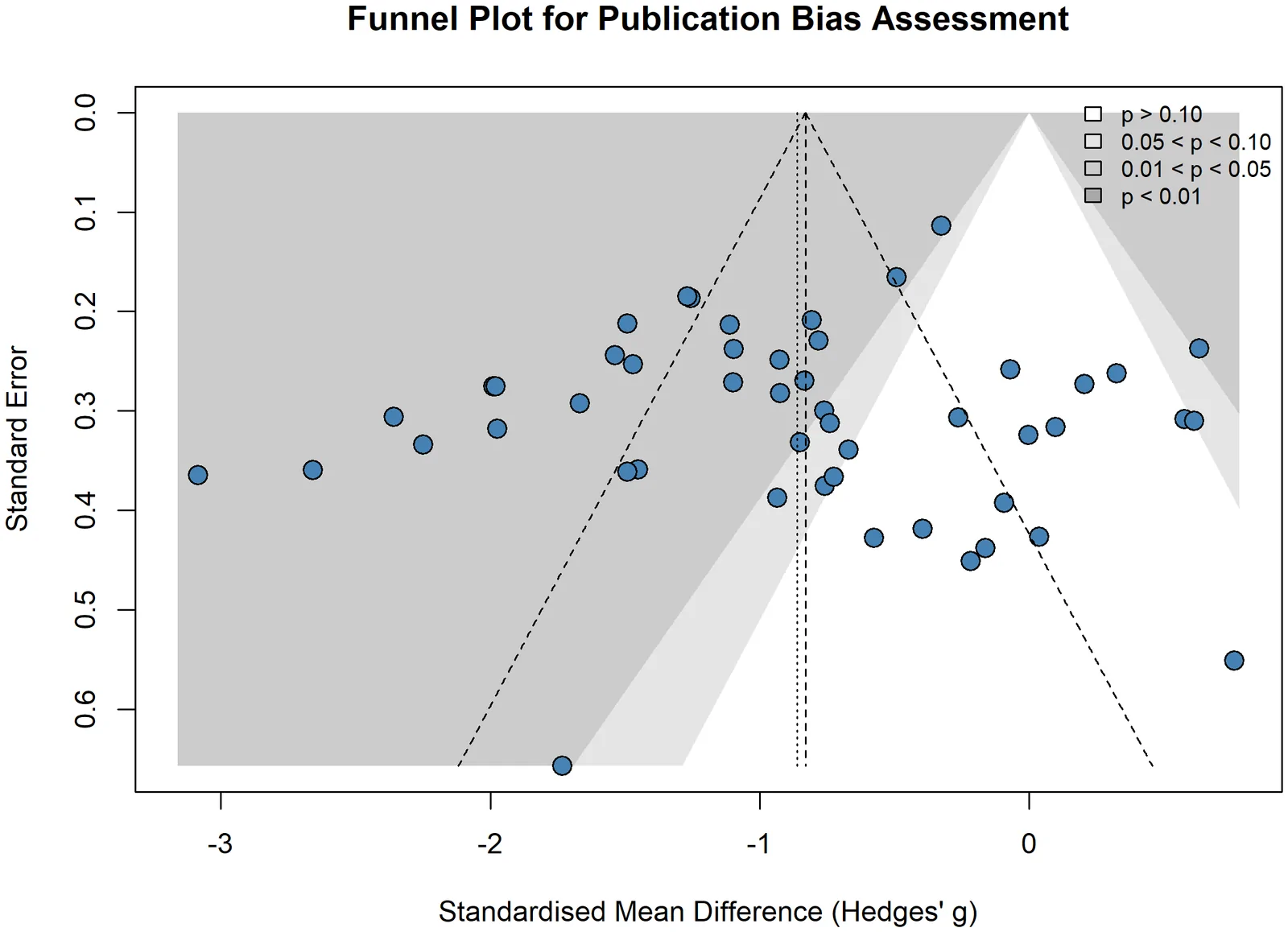
Funnel plot.

Potential publication bias was evaluated using a comparison-adjusted funnel plot. Visual inspection revealed a relatively symmetrical distribution of effect sizes around the zero effect line, indicating the absence of overt publication bias (**Figure 7**). This observation was statistically corroborated by Egger’s test (P = 0.667), confirming no significant publication bias across the entire network. Furthermore, assessments based on the Confidence in Network Meta-Analysis (CINEMA) framework revealed that the certainty of evidence for tDCS, HF-rTMS, TBS, and LF-rTMS in improving social cognition was rated as moderate. Conversely, the clinical evidence for immersive VR, 2D screen-based interventions, and embodied social robots was downgraded due to wide credible intervals (CrIs) in certain comparative nodes and potential methodological biases. Specifically, owing to the highly immersive nature and high-frequency real-time feedback inherent in digital interventions, implementing strict double-blind protocols (blinding of participants and personnel) was practically unattainable in most primary studies. This inherent methodological limitation consequently attenuated the certainty of evidence for these specific modalities (**Supplementary Table 3**).

## 4 Discussion

The present study represents the pioneering network meta-analysis to integrate non-invasive brain stimulation (NIBS) and digital interventions within a unified comparative framework. The primary objective of this study was to comprehensively evaluate and contrast the relative efficacy of these two principal therapeutic domains in ameliorating social cognitive deficits among individuals with autism spectrum disorder (ASD).

### 4.1 Main results

The present study included 50 randomized controlled trials encompassing 3,191 participants, utilizing the improvement of core social cognitive abilities in ASD as the primary outcome measure. Our findings revealed that, among all evaluated non-pharmacological interventions, tDCS emerged as the optimal modality for ameliorating social cognition in individuals with ASD. Furthermore, the transcranial magnetic stimulation (TMS) family of interventions demonstrated substantial advantages. Specifically, central neuromodulation techniques, including HF-rTMS, TBS, and LF-rTMS, all achieved therapeutic effects significantly superior to both TAU and Sham interventions in enhancing social cognition. The pooled effect sizes derived from the network pairwise comparisons provide robust evidence that these three magnetic stimulation-based neuromodulatory techniques are highly efficacious in addressing social cognitive deficits in ASD. In contrast, this study also objectively uncovers a notable phenomenon within the current landscape of ASD interventions. Although digital intervention technologies—represented by immersive VR, 2D screen-based platforms, and embodied social robots—have garnered significant attention in recent proof-of-concept studies and cutting-edge explorations, they did not exhibit the optimal therapeutic efficacy within the current network evidence framework. However, this finding should not be interpreted as a direct negation of the clinical efficacy of digital interventions; rather, it highlights their current therapeutic limitations and underscores the necessity for further methodological refinement.

In the cognitive habilitation of individuals with ASD, early intervention and the neurodevelopmental window are widely recognized as pivotal determinants of rehabilitative prognosis. Younger children, characterized by heightened endogenous neuroplasticity, typically derive more pronounced foundational benefits from various rehabilitative interventions (60). However, after adjusting for the age factor, the relative efficacy rankings and pooled effect sizes among the various modalities did not exhibit statistically significant shifts. tDCS and rTMS remained firmly at the top of the efficacy hierarchy, while solitary digital interventions continued to show a relative disadvantage. These results underscore that the superior therapeutic advantage of non-invasive brain stimulation (NIBS) is consistent across different age groups and is not merely an artifact of age-related baseline differences.

Importantly, these findings do not negate the critical value of the early neurodevelopmental window. Rather, they demonstrate that the substantial efficacy disparity observed—for instance, the pronounced therapeutic gains in older children receiving NIBS compared to the limited improvements in younger children receiving embodied social robots—is primarily dictated by the intrinsic properties of the interventions themselves, rather than being significantly moderated by chronological age. Specifically, neuromodulatory techniques such as rTMS and tDCS exert their effects by directly stimulating the cerebral cortex via targeted magnetic fields or electrical currents. The neuroplastic changes induced by such direct biophysical stimulation, particularly long-term potentiation (LTP), operate largely as a “bottom-up” mechanism. Consequently, this physiologically driven efficacy does not strictly depend on the patient’s subjective cognitive maturation, baseline developmental level, or active behavioral engagement.

### 4.2 Therapeutic mechanisms

To elucidate the disparities in therapeutic efficacy among the evaluated modalities, the mechanistic underpinnings of TMS, tDCS, and digital interventions are systematically delineated below.

#### 4.2.1 TMS-based interventions

The pronounced efficacy demonstrated by TMS techniques, namely HF-rTMS, LF-rTMS, and TBS, is fundamentally rooted in their capacity to directly modulate the core neuropathophysiological substrates of ASD, specifically targeting the aberrant connectivity within the social brain network (61). Deficits in higher-order social cognition among individuals with ASD, such as face processing and theory of mind, predominantly originate from an excitation/inhibition imbalance within critical cortical nodes, including the medial prefrontal cortex, temporoparietal junction, and superior temporal sulcus. Evidence indicates that applying HF-rTMS over the dorsolateral prefrontal cortex not only activates the localized cortical area but also orchestrates large-scale brain network remodeling (62). This high-intensity, “top-down” stimulation paradigm induces localized long-term potentiation at the targeted site while concurrently enhancing functional connectivity with distal brain regions via structural neural projections. Consequently, this mechanism substantially bolsters the brain’s capacity to decode complex social information (63). Furthermore, theta-burst stimulation mimics the endogenous theta rhythms naturally occurring in the hippocampus, delivering high-density pulses within a highly condensed timeframe. This rapid burst mechanism efficiently triggers the release of brain-derived neurotrophic factor (64), thereby accelerating the structural remodeling of synapses and the restoration of neuronal microcircuits (65).

#### 4.2.2 tDCS-based interventions

Typically delivering a weak direct current of 1 to 2 mA, tDCS is insufficient to directly elicit action potentials (66). Instead, it modulates the spontaneous firing rates of neurons by altering their resting membrane potentials (67). A pivotal neurobiological hallmark of ASD is the disruption of excitation/inhibition (E/I) homeostasis within the brain’s social cognitive network (68). By applying a constant, low-intensity direct current, tDCS induces polarity-dependent, subthreshold polarization of cortical neurons. This bidirectional modulatory capacity enables tDCS to precisely reconstruct the dynamic E/I balance across both localized cortical regions and interhemispheric networks. Furthermore, sustained stimulation upregulates the expression of brain-derived neurotrophic factor (BDNF), thereby inducing long-term potentiation (LTP)-like alterations in synaptic plasticity (69). Crucially, when tDCS is administered concurrently with conventional behavioral or cognitive rehabilitation, the applied current leverages the principles of Hebbian plasticity to specifically target and consolidate neural circuits that are actively engaged by the concurrent task (70).

#### 4.2.3 Based on digital intervention

Another pivotal finding of this study is that digital intervention technologies—represented by immersive VR, 2D screen-based interactions, and embodied social robots—ranked at the inferior end of the efficacy spectrum within the current evidence network. These technologies primarily rely on a “bottom-up” peripheral intervention pathway. By providing highly structured, safe, and replicable virtual social scenarios, they utilize multisensory feedback (including visual and auditory stimuli) in an attempt to activate the mirror neuron system (MNS) in individuals with ASD, thereby inducing observational learning and emotional empathy.

However, according to the well-established “broken mirror hypothesis” of ASD, the MNS in these patients inherently exhibits atypical anatomical and functional neurodevelopment (71). When the central nervous system remains in a state of functional impairment or blockaded connectivity, even the most high-definition and high-fidelity peripheral sensory inputs struggle to be efficiently translated into semantic comprehension and reciprocal social responses (72). Although digital technologies offer high ecological validity and cost-effectiveness, without the effective prior activation of the underlying central neural information integration networks, it is profoundly difficult for patients to progress from mere behavioral mimicry to spontaneous self-expression. In other words, in the absence of preceding central neural excitatory priming, solitary digital behavioral training is insufficient to trigger deep, enduring neuroplastic changes (73). Furthermore, this study uncovers a compelling phenomenon: the relative probability of efficacy for Sham stimulation was higher than that of embodied social robots and 2D screen interactions. It is crucial to note that the control groups in the included studies received Sham concurrently with treatment as usual (TAU). This outcome suggests that highly embodied robots or high-frequency screen interactions might impose excessive sensory overload or cognitive burden on children with ASD, who frequently present with sensory defensiveness, thereby triggering behavioral aversion or resistance. Conversely, the administration of Sham stimulation inevitably entails genuine, face-to-face interactions between researchers or healthcare professionals and the pediatric patients. Such non-specific social companionship, grounded in authentic human empathy, likely generates a more potent placebo effect—and therapeutic alliance—in improving social cognition than rigid, pre-programmed 2D interfaces.

### 4.3 Clinical implications

#### 4.3.1 Standalone neuromodulation protocols

Although tDCS attained the highest probability of efficacy in our statistical model, translating this finding into pediatric clinical practice necessitates a rigorous assessment of patient compliance. As a fundamentally mild intervention, tDCS typically induces only a transient, slight tingling sensation during the initial onset of stimulation. For young children with ASD, who frequently exhibit sensory defensiveness and profound auditory or tactile hypersensitivity, tDCS currently represents the most highly tolerated NIBS modality. Furthermore, the risk of tDCS-induced epileptogenesis is exceptionally negligible (74).

However, the mean age of the participants across the included trials was approximately 7 years. At this specific neurodevelopmental stage, pediatric patients with ASD universally present with short attention spans, hyperactivity, and a profound intolerance for unfamiliar or restrictive medical environments. Conventional tDCS protocols necessitate that patients remain immobilized in a treatment chair for 20 to 30 minutes. In real-world clinical execution, this prolonged physical restriction frequently provokes acute behavioral resistance or emotional dysregulation (meltdowns), inevitably driving up intervention dropout rates (75). In stark contrast, theta-burst stimulation (TBS) demonstrated robust clinical efficacy in our network while boasting a radically condensed single-session duration of approximately 3 minutes. This ultra-brief stimulation protocol perfectly accommodates the transient attention spans characteristic of pediatric patients, significantly lowering the threshold for behavioral compliance while simultaneously reducing the temporal burden on caregivers. Therefore, comprehensively weighing absolute therapeutic efficacy, sensory and temporal tolerability, and clinical throughput, this study proposes that tDCS and TBS should be broadly advocated as first-line translational neuromodulation strategies for ameliorating social cognitive deficits in ASD.

#### 4.3.2 Synergistic intervention paradigms

The current findings elucidate the limitations of immersive VR, 2D screen-based platforms, and embodied social robots when deployed as standalone interventions. However, this should by no means be construed as a negation of the core value of digital therapeutics in ASD rehabilitation. On the contrary, digital technologies possess unique attributes inherently lacking in traditional physical neuromodulation devices, specifically high ecological validity, robust safety profiles, and the capacity to reliably reproduce highly structured social scenarios. As demonstrated, relying exclusively on the “bottom-up” paradigm of digital interventions fails to adequately activate the patient’s underlying central neural information integration networks, rendering it exceedingly difficult to induce transformative, long-term improvements in social cognitive functioning.

Building upon this premise, future clinical practice for ASD should transcend the dichotomous “either-or” dilemma between non-invasive brain stimulation (NIBS) and digital therapeutics. Instead, a synergistic treatment paradigm integrating NIBS with digital interventions must be established. Specifically, this paradigm could comprise two tightly coupled interventional phases. Initially, rTMS is utilized to stimulate the dorsolateral prefrontal cortex (dlPFC) or the temporoparietal junction (TPJ), upregulating cortical excitability within the social brain network and thereby opening a critical window of neuroplasticity. Subsequently, within this primed neuroplastic window, high-cognitive-load immersive VR social interactions or embodied social robotic tasks are introduced. Alternatively, such digital interventional strategies can be integrated concurrently during the administration of tDCS. By leveraging neuromodulation to lower the threshold for synaptic modification, while simultaneously harnessing digital therapeutics to deliver precise multisensory feedback, this synergistic strategy ensures that every positive reinforcement derived from behavioral training is firmly consolidated within the already-activated central neural networks. Ultimately, this approach promises a substantially more efficacious amelioration of core social cognitive deficits in ASD.

### 4.4 Limitations

①the inherent nature of non-pharmacological interventions precluded strict double-blinding in most primary studies. ②although we strived to ensure clinical homogeneity by strictly extracting social cognition-specific sub-scores, utilizing diverse psychometric instruments (e.g., CARS, SRS, ABC) across trials inevitably introduces intrinsic variances in measurement sensitivity. Because stratifying the network by specific scale types would lead to fragmented networks and compromise statistical power, we were unable to conduct scale-specific sensitivity analyses to parse out this residual heterogeneity. ③despite no overt bias detected by funnel plots or Egger’s test, the non-publication of negative results poses a residual risk of publication bias that could subtly skew pooled effect estimates. ④relying on aggregate mean ages for meta-regression, rather than individual participant data (IPD), carries a risk of ecological fallacy. Furthermore, this reliance on aggregated data precluded us from conducting categorical subgroup analyses based on distinct age strata (e.g., preschool-aged vs. adolescents), which limits our ability to fully validate the indirect comparisons across different neurodevelopmental stages. Future IPD-based research is warranted to elucidate individual-level therapeutic nuances across different neurodevelopmental stages. ⑤The included primary studies generally lacked detailed reporting on additional disabilities or comorbidities (e.g., intellectual disability or ADHD), which limits our ability to evaluate the potential moderating effects of these co-occurring conditions on therapeutic outcomes.

## 5 Conclusion

tDCS emerges as the most efficacious standalone intervention for ameliorating social cognitive deficits in ASD, significantly outperforming single-modality digital therapies. Clinically, we recommend prioritizing tDCS or TBS. Ultimately, we advocate a synergistic therapeutic paradigm that integrates NIBS-induced neural priming with high-ecological-validity digital training. Large-scale RCTs remain essential to validate these combined protocols.

## Data Availability

The original contributions presented in the study are included in the article/**Supplementary Material**. Further inquiries can be directed to the corresponding authors.
