# Supplementary material for: The impact of non-invasive brain stimulation and digital intervention on social cognitive therapy in autism spectrum disorder: a systematic review and network meta-analysis
Source: Front Psychiatry. 2026 Aug 31;17:1877176. doi: 10.3389/fpsyt.2026.1877176 (PMC13572590; doi:10.3389/fpsyt.2026.1877176)
Supplement: Supplementary file 1 [file DataSheet1.zip › Supplementary files/Supplementary Table 1.docx]

Table 1. Basic characteristics of the included studies

| **Study** | **Sample Size** | | **Age (Years)** | | **Outcome Measures** |
| --- | --- | --- | --- | --- | --- |
|  | **Intervention** | **Comparator** | **Intervention** | **Comparator** |  |
| Zhao 2021 | 57 | 57 | 3-5 | 3-5 | ATEC,ABC,PEP-3,CARS |
| Ye 2020 | 32 | 32 | 3.51±1.03 | 3.51±1.03 | Gesell,ABC,CARS |
| Ni 2021 | 40 | 40 | 4.05±0.44 | 4.05±0.44 | CSHQ,CARS,ABC |
| Zhang 2022 | 30 | 30 | 12.9±1.62 | 13.5±2.31 | ABC |
| Guan 2023 | 40 | 40 | 3.71±1.05 | 3.76±1.01 | ABC,CARS |
| Yang 2021 | 17 | 14 | 8.15±1.78 | 9.43±2.38 | ABC |
| Liu 2019 | 20 | 20 | 3.50±0.20 | 3.45±0.25 | ABC,CARS,DQ |
| Chen 2021 | 20 | 20 | 5.26±1.21 | 5.34±1.24 | CARS,ABC,GDS-C,plasma biochemical indicators |
| Wu 2016 | 25 | 23 | 4.14±1.25 | 4.28±1.19 | ABC,CARS,DQ |
| Shao 2024 | 51 | 51 | 5.48±0.86 | 5.63±0.79 | ATEC,CARS,ABC,SRS,CSHQ |
| Guo 2023 | 69 | 69 | 3-5 | 3-5 | ABC,SCARED,DSRSC,WISC,CSIRS |
| Wang 2022 | 37 | 37 | 6.25±0.61 | 6.32±0.51 | CARS,ATEC,BDNF,DA,DQ |
| Wang 2023 | 41 | 41 | 5.58±1.31 | 5.63±1.40 | ABC,CARS,CSHQ |
| Liu 2024 | 72 | 69 | 2-6 | 2-6 | ABC,CARS,VB-MAPP |
| Huang 2024 | 82 | 69 | 3.80±1.30 | 3.60±3.02 | CARS,CSCL |
| LI 2024 | 16 | 16 | 3.19±0.77 | 2.83±0.71 | ABC,CARS,RBS-R,ATEC, Resting-state fMRI |
| Zhang 2024 | 30 | 30 | 12.11±1.68 | 11.56±1.76 | ATEC,PedsQL4.0 |
| Ma 2024 | 41 | 41 | 5.45±1.58 | 5.85±1.45 | ABC,CARS,CSIRS |
| Nu 2025 | 40 | 40 | 4.11±0.56 | 4.16±0.64 | CARS,ABC,PSQI,ATEC |
| Hu 2025 | 46 | 54 | 3.83±2.21 | 4.76±1.13 | ATEC,ABC,WeeFIM,CARS,CFCS |
| Qiu 2025 | 31 | 32 | 3.96±0.68 | 3.76±0.74 | ABC,CARS,CSHQ,DQ |
| Dou 2018 | 30 | 30 | 3-4 | 3-4 | ABC,CARS |
| Guo 2025 | 28 | 28 | 4.83±0.99 | 4.96±0.96 | ABC,CARS,SDQ,SRS |
| Shi 2026 | 44 | 44 | 3-6 | 3-6 | PEP-3,ATEC |
| Wu 2026 | 20 | 20 | 8.65±1.79 | 8.70±1.89 | CARS, fMRI degree centrality |
| Huang 2024 | 34 | 34 | 9.60±0.50 | 9.70±0.60 | SIS,C-PEP,ABC,EEG |
| Qiu 2021 | 20 | 20 | 52.25±14.71month | 52.10±15.65month | CARS,ABC,RBS-R,CSHQ |
| Sun 2022 | 19 | 18 | 8.0±1.9 | 7.6±2.1 | ABC, EEG MMN features |
| Ip 2018 | 36 | 36 | 107.60±13.27month | 104.80±13.83month | PEP-3,Face test, eye contact test,ABAS-II |
| Liu 2025 | 155 | 157 | 5.1±1.7 | 5.1±1.5 | ATEC,ABC,CSHQ |
| Luckhardt 2025 | 10 | 11 | 14.01±2.66 | 13.82±2.57 | SRS,RBS-R,CSCL,CCC2-R,ERP |
| Amatachaya 2014 | 20 | 20 | 6.30±1.10 | 6.30±1.10 | CARS,ATEC,CGAS,CGI-I |
| Zhao 2022 | 22 | 22 | 3.45(3.00-4.00) | 3.50(3.00-4.00) | PEP-3 |
| Lee 2023 | 9 | 6 | 9.0(8.0-10.0) | 8.5(8.0-10.0) | SRS-2,K-SCQ,K-CSCL,K-VABS-2,SCQ |
| Jouen 2017 | 14 | 10 | 6.85±1.34 | 7.17±1.62 | ADOS,VABS-II,CSCL,SCQ,PSI |
| Holeva 2022 | 22 | 22 | 9.68±1.87 | 9.27±2.01 | CARS-2,NEPSY-II,ASEBA(CSCL/TRF),SDQ |
| Fletche 2016 | 27 | 27 | 49.30±10.9month | 49.96±13.2month | ADOS-2,BOSCC,MCDI,CSBS-DP,MSEL |
| De 2020 | 24 | 20 | 3-8 | 3-8 | SRS |
| Marino 2020 | 7 | 7 | 73.3±16.1month | 82.1±12.4month | TEC,ELT |
| Ni 2023 | 30 | 30 | 13.1±3.4 | 13.2±3.2 | SRS,RBS-R,EDI,ABAS-II,RMET, Frith-Happe Animation Mission,RPM,BRIEF |
| Wang 2023 | 15 | 15 | 7.33±3.31 | 7.26±2.94 | SRS |
| Prillinger 2024 | 11 | 11 | 14.00±1.90 | 14.27±1.90 | ERT,SRS |
| Ni 2017 | 19 | 19 | 20.8±1.4 | 20.8±1.4 | CCPT,WCST,Y-BOCS,SRS |
| Ni 2024 | 13 | 13 | 12.9±6.7 | 13.1±6.3 | EDI,ABC-I,SRS,RBS-R,ABAS-II |
| Tan 2025 | 30 | 30 | 7.06±1.55 | 7.06±1.55 | SRS,CCDI,PPVT,MAIN,CGI-I |
| Whitehouse 2017 | 39 | 36 | 39.36±8.50month | 40.25±8.41month | ATEC,MSEL,VABS-II,MCDI,CSBS,RBS-R,BFRS-R |
| Zheng 2020 | 11 | 9 | 2.55±0.45 | 2.54±0.40 | STAT |
| Thomeer 2015 | 22 | 21 | 8.86±1.39 | 8.57±1.16 | CAM-C,ERDS,SRS,BASC-2 |
| Parsons 2018 | 30 | 29 | 64.40±22.73month | 60.80±15.47month | MSEL,SPT,CSBS,ToP,POM |
| So 2019 | 12 | 11 | 5.62±1.29 | 5.49±1.35 | ESCS,SPA,SRS |

*：CARS Childhood Autism Rating Scale；ABC Autism Behavior Checklist；ATEC Autism Treatment Evaluation Checklist；SRS Social Responsiveness Scale；ADOS Autism Diagnostic Observation Schedule；PEP-3 Psychoeducational Profile-Third Edition；SCQ Social Communication Questionnaire；RBS-R Repetitive Behavior Scale-Revised；BOSCC Brief Observation of Social Communication Change；ESCS Early Social Communication Scales；STAT Screening Tool for Autism in Toddlers and Young Children；CSBS Communication and Symbolic Behavior Scales；VABS-II Vineland Adaptive Behavior Scales-Second Edition；ABAS-II Adaptive Behavior Assessment System-Second Edition；Gesell Gesell Developmental Schedules；DQ Developmental Quotient；WISC Wechsler Intelligence Scale for Children；MSEL Mullen Scales of Early Learning；RPM Raven's Progressive Matrices；NEPSY-II A Developmental NEuroPSYchological Assessment-Second Edition；VB-MAPP Verbal Behavior Milestones Assessment and Placement Program；WeeFIM Functional Independence Measure for Children；CBCL Child Behavior Checklist；ASEBA Achenbach System of Empirically Based Assessment；SDQ Strengths and Difficulties Questionnaire；CSHQ Children's Sleep Habits Questionnaire；PSQI Pittsburgh Sleep Quality Index；SCARED Screen for Child Anxiety Related Emotional Disorders；DSRSC Depression Self-Rating Scale for Children；BRIEF Behavior Rating Inventory of Executive Function；Y-BOCS Yale-Brown Obsessive Compulsive Scale；CGAS Children's Global Assessment Scale；CGI-I Clinical Global Impression-Improvement；PedsQL 4.0 Pediatric Quality of Life Inventory Version 4.0；PSI Parenting Stress Index；SIS Sensory Integration Scale；TEC Test of Emotion Comprehension；RMET Reading the Mind in the Eyes Test；ERT Emotion Recognition Task；CCPT Conners' Continuous Performance Test；WCST Wisconsin Card Sorting Test；SPA Structured Play Assessment；SPT Symbolic Play Test；MCDI MacArthur-Bates Communicative Development Inventories；CCDI Chinese Communicative Development Inventory；CCC2-R Children's Communication Checklist-2；PPVT Peabody Picture Vocabulary Test；BDNF Brain-Derived Neurotrophic Factor；DA Dopamine；fMRI Functional Magnetic Resonance Imaging；DC Degree Centrality；ERP Event-Related Potential；MMN Mismatch Negativity.
