# Supplementary material for: The impact of non-invasive brain stimulation and digital intervention on social cognitive therapy in autism spectrum disorder: a systematic review and network meta-analysis
Source: Front Psychiatry. 2026 Aug 31;17:1877176. doi: 10.3389/fpsyt.2026.1877176 (PMC13572590; doi:10.3389/fpsyt.2026.1877176)
Supplement: Supplementary file 1 [file DataSheet1.zip › Supplementary files/Supplementary Table 2.docx]

Table 2. Intervention measures included in the literature

| **Study** | **Intervention** | | **Parameters** | **Adverse Events** |
| --- | --- | --- | --- | --- |
|  | **Intervention** | **Comparator** |  |  |
| Zhao 2021 | VR technology + TAU | TAU | 2 sessions/week, 20 min/session; total 6 months | Not explicitly reported |
| Ye 2020 | Virtual Reality Social Skills Training (VR-SST) | TAU | 3 sessions/week, 30 min/session; total 3 months | Not explicitly reported |
| Ni 2021 | LF-rTMS + TAU | TAU | rTMS 1 session/day for 10 days/course; rehab 5 sessions/week; total 4 months | Not explicitly reported |
| Zhang 2022 | LF-rTMS + TAU | TAU | rTMS 5 sessions/week; routine intervention 5 days/week; total 6 months | Explicitly reported "no adverse reactions observed" |
| Guan 2023 | iTBS + Sandplay therapy + TAU | TAU | iTBS 3 sessions/day; sandplay 1 session/day; total duration 8 weeks | Not explicitly reported |
| Yang 2021 | iTBS (target STS) + TAU | TAU | iTBS 3 sessions/day; rehab 5 days/week; total 4 weeks (28 days) | 1 case of mild headache, 2 cases of transient excitation in exp. group; no severe adverse events |
| Liu 2019 | Ultra-low frequency TMS + TAU | TAU | 1 session/day for first 10 days, 2 sessions/week for next 2 weeks, 1 session/week for last 3 weeks; total approx. 6 weeks | Not explicitly reported |
| Chen 2021 | LF-rTMS + TAU | TAU | rTMS 5 days/week; comprehensive training 1 session/day; total 6 months | Not explicitly reported |
| Wu 2016 | HF-rTMS + TAU | TAU | Magnetic stimulation 20 min/session, 10 days/course; total 4 months | Not explicitly reported |
| Shao 2024 | LF-rTMS + TAU | TAU | rTMS 1 session/day, 5 days/week; routine sandplay intervention; total duration unspecified | Not explicitly reported |
| Guo 2023 | Anodal tDCS + TAU | Sham + TAU | Total 6 weeks | No obvious adverse reactions observed in either group during treatment |
| Wang 2022 | HF-rTMS + TAU | TAU | rTMS 1 session/day, 5 days/week; total 2 months | Not explicitly reported |
| Wang 2023 | LF-rTMS + TAU | TAU | rTMS 1 session/day, 5 days/week; total 8 weeks | Not explicitly reported |
| Liu 2024 | HF-rTMS + TAU | TAU | rTMS 1 session/day, 5 days/week; total 3 months (3 courses) | Not explicitly reported |
| Huang 2024 | LF-rTMS + Applied Behavior Analysis (ABA) | TAU | rTMS 1 session/day, 5 days/week for 4 weeks; total intervention 16 weeks | No seizures or severe adverse events observed |
| LI 2024 | LF-rTMS + TAU | TAU | rTMS 1 session/day, 5 days/week; total 12 weeks | Not explicitly reported |
| Zhang 2024 | LF-rTMS + TAU | TAU | rTMS 1 session/day, 5 sessions/week; total duration 6 weeks | Not explicitly reported |
| Ma 2024 | LF-rTMS + Sensory Integration Training + TAU | TAU | rTMS 1 session/day, 6 days/week; total duration 3 months | Not explicitly reported |
| Nu 2025 | HF-rTMS + Sensory Integration Training + TAU | TAU | rTMS 5 sessions/week, 20 min/session; total duration 3 months | Not explicitly reported |
| Hu 2025 | Pivotal Response Treatment (PRT) + LF-rTMS | TAU | rTMS 1 session/day, 5 days/week; PRT at least 3 sessions/week; total duration 8 weeks | Not explicitly reported |
| Qiu 2025 | Multisensory Training + LF-rTMS | TAU | Total duration 8 weeks | No statistical difference in incidence rate; specific data not reported |
| Dou 2018 | Ultra-low frequency (ILF-TMS) + ABA | TAU | Magnetic stimulation 1 session/day, ABA 5 days/week; total 12 weeks | Not explicitly reported |
| Guo 2025 | VR Rehab Training + Early family intervention | TAU | VR training 1 session/week, 1 h/session; total 2 months | Not explicitly reported |
| Shi 2026 | VR-based Rehab Training + TAU | TAU | VR 1 session every 2 days, 15 min/session; total 12 weeks | Not explicitly reported |
| Wu 2026 | Left dlPFC LF-rTMS (1Hz) + TAU | Sham + TAU | rTMS 5 sessions/week, 180 pulses/session; total 4 weeks | Not explicitly reported |
| Huang 2024 | tDCS + Sensory Integration Training | Sham + TAU | Sensory integration 5 sessions/week, 60 min/session; total 6 weeks | Not explicitly reported |
| Qiu 2021 | Left dlPFC Anodal tDCS + TAU | Sham + TAU | 5 sessions/week, 20 min/session; total 3 weeks + 1 | 1 case of hyperactivity in exp. group, resolved 1 week post-stimulation; no severe AEs |
| Sun 2022 | Left dlPFC Anodal tDCS + TAU | Sham + TAU | 3 sessions/week, 20 min/session; total 4 weeks + 1 | Mild tingling at stimulation onset; no severe adverse reactions reported |
| Ip 2018 | Immersive Virtual Reality (VR) System Training | TAU | Total 28 sessions; duration 14 weeks | Not explicitly reported |
| Liu 2025 | tPCS + TAU | Sham + TAU | 1 session/day, 20 min/session; total 20 sessions (4 weeks) | Mild headache and local skin redness; no severe adverse events |
| Luckhardt 2025 | Bilateral TPJ Anodal tDCS + Cognitive Training | Sham + TAU | 20 min/session; total 10 sessions (2 weeks) | Stimulation well tolerated; no severe adverse events |
| Amatachaya 2014 | Anodal tDCS (target F3) | Sham + TAU | 1 session/day, 20 min/session; 5 consecutive days | Some in exp. group reported transient mild headache/deterioration |
| Zhao 2022 | TAU + VR Technology Training | TAU | 3 sessions/week, 15 min/session; total 12 weeks | Initially, some children refused VR headsets ("desensitization therapy" applied) |
| Lee 2023 | Metaverse-based (Roblox+Zoom) VR Social Skills Training | TAU | 1 session/week, 60 min/session; total 4 weeks | No adverse events related to study participation |
| Jouen 2017 | GOLIAH serious game platform + TAU | TAU | 4 home sessions (30 min each) + 1 hospital session/week; total 6 months | Parenting Stress Index (PSI) unchanged; 3 children showed very low engagement |
| Holeva 2022 | Robot (NAO)-assisted psychological intervention | TAU | 2 sessions/week, 35-45 min/session; total 21 sessions (approx. 3 months) | Withdrawals due to group dissatisfaction/medical issues; no AEs directly induced by intervention |
| Fletche 2016 | iPad application (FindMe) + TAU | TAU | ~5 min/day or 10 min every other day; total 2 months | Lack of harms explicitly stated; 1 participant withdrew due to disliking intervention |
| De 2020 | Robot (NAO)-assisted Pivotal Response Treatment (PRT) | TAU | 1 session/week, 45 min/session; total 20 weeks | No obvious adverse events reported in the text |
| Marino 2020 | Embodied Social Robot (NAO)-assisted Group CBT | TAU | 2 sessions/week, 90 min/session; total 10 sessions (excluding 2 assessments) | 1 child excluded during assessment phase due to aggressive behavior |
| Ni 2023 | Left dlPFC continuous theta-burst stimulation (cTBS) | Sham + TAU | 600 pulses/session (~40 sec), 2 sessions/week, 8 consecutive weeks (16 sessions) | Safe/feasible. Evaluated pain, headache/dizziness, tinnitus; no severe AEs reported |
| Wang 2023 | 2mA Cz-centered Anodal High-Definition tDCS (HD-tDCS) | Cz Sham + TAU | 20 min/session, 5 sessions/week, 3 consecutive weeks (14 sessions) | Well tolerated, no severe AEs. 21 children in exp. group felt pain at central anode initially |
| Prillinger 2024 | 2mA Anodal tDCS (F3/right supraorbital) + Online Emotion Recognition | Sham + TAU | 20 min/session, 10 sessions over 2 consecutive weeks | High tolerance/acceptability in both groups; no severe adverse reactions reported |
| Ni 2017 | Single-session iTBS over bilateral dlPFC or pSTS | Sham + TAU (Inion) | Single stimulation per target (1-week interval), 600 pulses/hemisphere | 3 reported transient periocular muscle twitching discomfort during dlPFC stim; no headache/seizures |
| Ni 2024 | iTBS over left dlPFC | Sham + TAU | 5 sessions/week, 4 consecutive weeks (20 sessions), 600 pulses/session | Exp. group had mild transient local pain (38%) & dizziness (8%). No severe AEs, no dropouts |
| Tan 2025 | Accelerated cTBS (a-cTBS) targeting left M1 | Sham + TAU | 10 sessions/day, 5 consecutive days (50 sessions), 1800 pulses/session | 10 (33%) had mild AEs: irritability (n=6), scalp pain (n=3), nausea (n=1), all resolved spontaneously |
| Whitehouse 2017 | TOBY iPad app-assisted home training + TAU | TAU | Recommended ≥20 min/day, 6 consecutive months | High dropout rate; withdrawals due to app disinterest (n=1) or audiovisual hypersensitivity |
| Zheng 2020 | Intelligent closed-loop robot (NAO)-mediated joint attention intervention | TAU | 4 robot sessions, ~10 min/session (spanning 3-9 weeks) | 3 children (1 exp., 2 control) withdrew due to evident distress and discomfort initially |
| Thomeer 2015 | Emotion recognition software (MindReading) + In-vivo role-play + Reinforcement | TAU | 2 sessions/week, 90 min/session, 12 consecutive weeks (24 sessions) | 1 control child excluded for psychiatric symptoms; no direct AEs from intervention reported |
| Parsons 2018 | TOBY iPad app + TAU | TAU | Recommended ≥20 min/day, 3 consecutive months | 3 withdrew as "child unable to engage/disinterested"; actual avg. use 11.3 min/day (low compliance) |
| So 2019 | Embodied Social Robot (NAO)-based drama play + role-play | TAU | 1 session/week, 45 min/session, covering 3 plays, 9 consecutive weeks (9 sessions) | Adverse reactions not explicitly reported in the text |
