# Supplementary material for: The impact of non-invasive brain stimulation and digital intervention on social cognitive therapy in autism spectrum disorder: a systematic review and network meta-analysis
Source: Front Psychiatry. 2026 Aug 31;17:1877176. doi: 10.3389/fpsyt.2026.1877176 (PMC13572590; doi:10.3389/fpsyt.2026.1877176)
Supplement: Supplementary file 1 [file DataSheet1.zip › Supplementary files/Supplementary Table 3.docx]

| Comparison vs TAU | Within-study Bias | Reporting Bias | Indirectness | Imprecision | Heterogeneity | Incoherence | Certainty |
| --- | --- | --- | --- | --- | --- | --- | --- |
| HF-rTMS | Downgraded 1 level a | Downgraded 1 level a | Downgraded 1 level a | Downgraded 1 level a | Downgraded 1 level a | Downgraded 1 level a | Moderate |
| TBS | Downgraded 1 level a | Downgraded 1 level a | Downgraded 1 level a | Downgraded 1 level a | Downgraded 1 level a | Downgraded 1 level a | Moderate |
| LF-rTMS | Downgraded 1 level a | Downgraded 1 level a | Downgraded 1 level a | Downgraded 1 level a | Downgraded 1 level a | Downgraded 1 level a | Moderate |
| Immersive VR | Downgraded 1 level a | Downgraded 1 level a | Downgraded 1 level a | Downgraded 1 level b | Downgraded 1 level a | Downgraded 1 level a | Low |
| 2D Screen-based | Downgraded 1 level a | Downgraded 1 level a | Downgraded 1 level a | Downgraded 1 level b | Downgraded 1 level a | Downgraded 1 level a | Low |
| Embodied Social Robots | Downgraded 1 level a | Downgraded 1 level a | Downgraded 1 level a | Downgraded 1 level b | Downgraded 1 level a | Downgraded 1 level a | Low |
